# Pelvic dystopia of right rudimentary multicystic dysplastic kidney as a rare cause of bedwetting in a patient with a single pelvic ectopic left kidney, and agenesis of the uterus and vagina (Mayer-Rokitansky-Küster-Hauser syndrome): a case report

**DOI:** 10.1186/s13256-018-1644-9

**Published:** 2018-05-07

**Authors:** Kidirali Karimbayev, Nazarbek Dzumanazarov, Mukhtar Akhaibekov, Nurzhan Berdikulov, Abay Karimbayev, Assanaly Mustafayev

**Affiliations:** 1 Department of Surgery, Akhmet Yassawi International Kazakh-Turkish University Medical School, Turkestan, Kazakhstan; 2 Pathology Department, Akhmet Yassawi International Kazakh-Turkish University, Medical School, Turkestan, Kazakhstan; 3 AkhmetYassawi International Kazakh-Turkish University, Clinical Diagnostic Center, Turkestan, Kazakhstan; 4 ENKA Company, Turkestan, Kazakhstan

**Keywords:** Bedwetting, Congenital causes, Pelvic dystopia of the rudimentary multicystic dysplastic kidney

## Abstract

**Background:**

Pelvic dystopia of rudimentary multicystic dysplastic kidney as a rare cause of bedwetting in children.

**Case presentation:**

We report the case of a 14-year-old Kazakh girl who presented with difficulty in starting the stream of urine and intermittent interruption of the urinary stream while voiding as well as bedwetting, caused by a rare congenital disease (pelvic dystopia of rudimentary multicystic dysplastic kidney). The diagnostic workup, differential diagnosis, and management, and a review of the literature are presented. Persistent since she was 2 years old, bedwetting was stressful for both the parents and child. Initially detected radiologically and endoscopically, a bladder mass was thought suspicious for ureterocele, papilloma, or mixed tumor of the urinary bladder, but surprisingly, turned out to be a pelvic dystopia of the rudimentary multicystic dysplastic kidney. Transvesical excision of this mass was performed.

**Conclusions:**

The purpose of this case report is to draw attention to the fact that a persistent case of bedwetting which does not respond to conventional therapy should be subject to further examinations to exclude surgical causes of the disease.

## Background

Nocturnal enuresis (bedwetting) is defined by the National Institute for Health and Care Excellence (NICE) guidelines as the involuntary wetting during sleep without any inherent suggestion of frequency of bedwetting or pathophysiology [1]. Bedwetting prevalence is 1.5–10% of 10-year-olds [2].

We report a rare case of a 14-year-old Kazakh girl with persistent bedwetting which did not respond to conventional therapy for a long period of time. This case was a diagnostic and therapeutic challenge for us because diagnostic radiology and endoscopy were suspicious for ureterocele, papilloma, or mixed tumor of the urinary bladder. In this case, only through a surgical procedure and histological examination was the true cause of bedwetting found. Therefore, good collaboration between surgeons and pathologists is essential for an accurate diagnostic process.

In such cases of obscure bladder mass that cause persistent bedwetting, transvesical excision is preferable.

We searched for information in sources such as Medline, Embase, and the Cochrane Database of Systemic Reviews using search terms causes of “enuresis” and “bedwetting” as keywords and did not find any reports apprising that dystopia of rudimentary multicystic dysplastic kidney can be the cause of bedwetting.

## Case presentation

A 14-year-old Kazakh girl who had ongoing difficulties in starting the stream of urine and intermittent interruption of the urinary stream while voiding, and bedwetting since her early years (persistent since she was 2 years old), which was stressful for both the parents and child.

Since that time, the girl has been treated by pediatricians using traditional methods (fluid and diet restriction, lifting and waking, dry bed training, bladder training and retention control, anticholinergic medication) without any success.

Her past medical history included regular medical checkups because of agenesis of her right kidney and pelvic ectopy of the single left kidney, a cyst of the bladder, and bedwetting. She had never had hematuria. Surgical interventions had not taken place. She is the second child of five in her family. The other family members do not have health problems.

On physical examination she was well. Her blood pressure was 100/60 mmHg. Her pulse was 82 beats/min. A urine analysis was normal. Her renal function was normal with a creatinine of 90 μmol/L. Examination of other systems revealed an agenesis of her vagina.

**Fig. 1:**
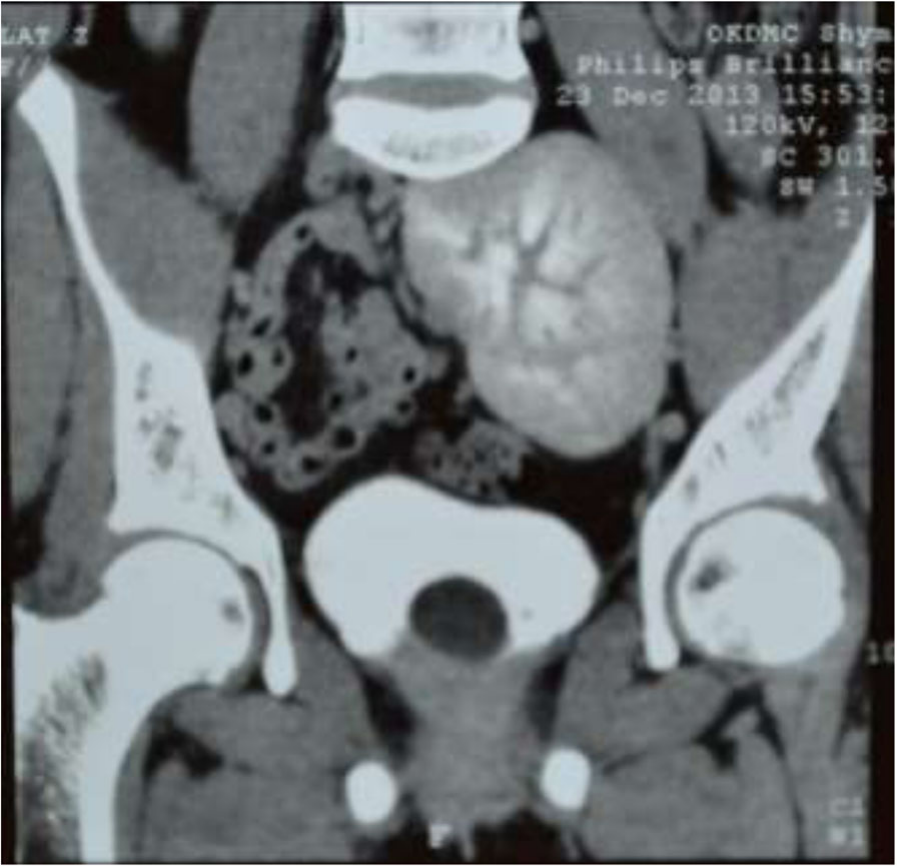
A contrast media computed tomography scan shows agenesis of the right kidney. Pelvic ectopy of the left kidney, 51.8 × 62.8 in size; on the right side of the urinary bladder there is a cystic mass 24 × 17 mm in size (papilloma, ureterocele, mixed tumor). Agenesis of uterus and vagina (Mayer-Rokitansky-Küster-Hauser syndrome) are shown

On radiologic examination: on sonography her right kidney was not visualized; her left kidney was located in the pelvic region, and was 51.8 × 62.8 cm in size. Her urinary bladder volume was 156 cm^3^, and on the right side of the wall a cystic mass 23 × 20 mm in size was visualized (ureterocele?). A contrast media computed tomography (CT) scan revealed agenesis of her right kidney, pelvic ectopy of her left kidney; on the right side of the urinary bladder there was a cystic mass 24 × 17 mm in size (papilloma, ureterocele, mixed tumor); and agenesis of her uterus and vagina (Mayer-Rokitansky-Küster-Hauser syndrome) was also detected (Fig. 1).

**Fig. 2:**
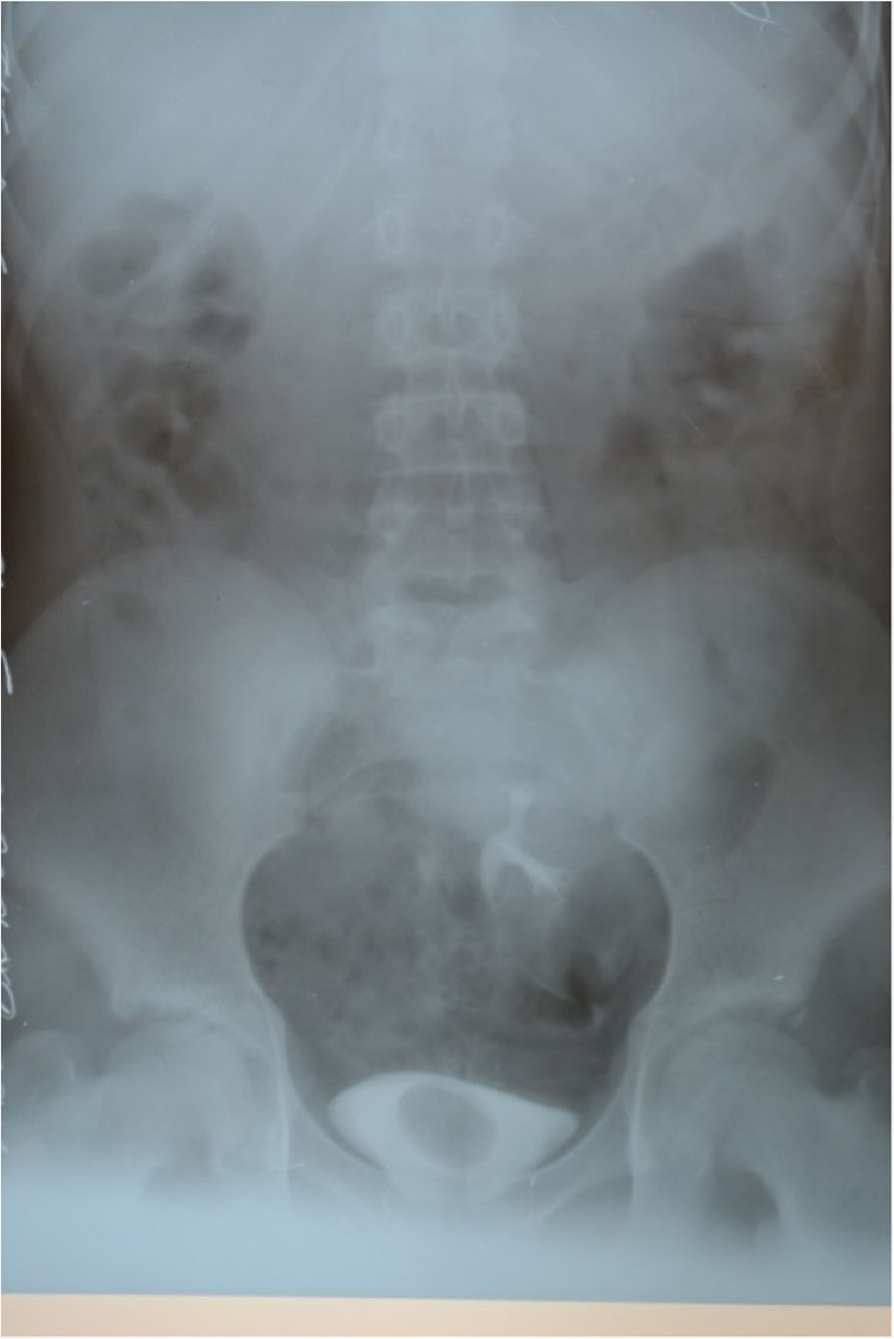
On intravenous urography the right kidney is not visualized, there was normal functioning of the left kidney located in the pelvic region, and a descending cystogram shows a shadow of reduced density mass on the right side of the urinary bladder wall, 23 × 20 mm in size (cystic mass, ureterocele, papilloma)

On intravenous urography, her right kidney was not visualized, there was normal functioning of the left kidney located in the pelvic region, and excretory (descending) cystography shows a shadow of reduced density on the right side of the urinary bladder wall 23 × 20 mm in size (cystic mass, ureterocele) (Fig. 2).

### Cystoscopy

On the right extremity of the interureteric ridge, in the projection of the missing right ureteric orifice, a round-shaped cystic mass, 24 × 20 mm in size was seen, covered by a normal mucous membrane, located close to the bladder neck and protruding into the internal urethral meatus.

Based on the clinical presentation, sonographic, radiologic and endoscopic findings we concluded that some cystic masses (mixed tumor, dermoid cyst) of the bladder cause bladder irritation with symptoms of lower urinary tract obstruction and bedwetting. However, we considered the possibility of a relationship of this mass with the right kidney agenesis, because of its location in the same area of the missing right ureteric orifice, so we decided to perform transvesical surgery rather than transurethral surgery.

### Procedure (May 14, 2014)

**Fig. 3:**
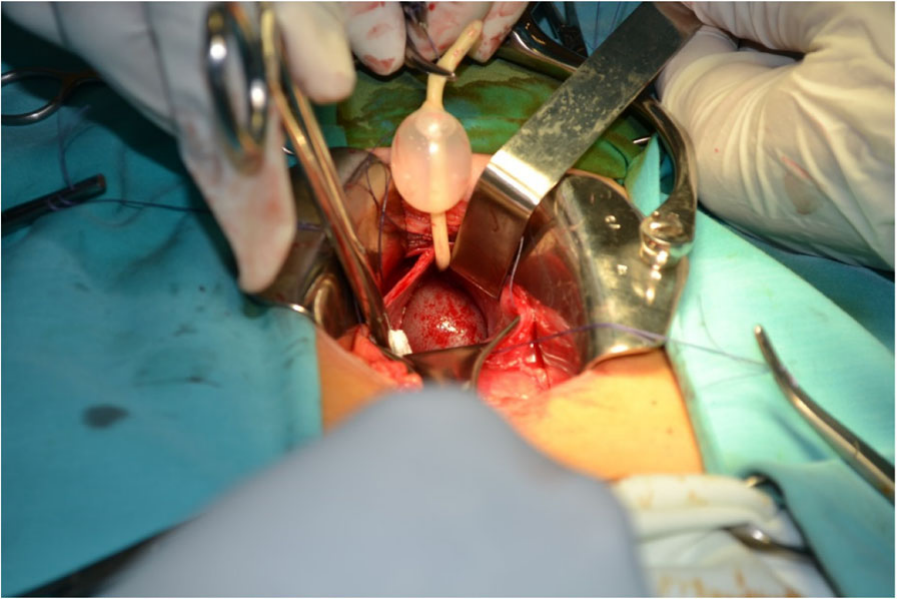
Transvesical views – a round-shaped mass, 24 × 20 mm in size, located on the right extremity of the interureteric ridge, covered by normal mucous membrane of the bladder. No right ureteric orifice seen. The mass was located close to the bladder neck and protruding into the internal urethral meatus. (*Ballooned urethral Foley catheter seen*)

Transvesical access found a round-shaped mass, 24 × 20 mm in size, located on the right extremity of the interureteric ridge, covered by normal mucous membrane of the bladder. This mass was located close to the bladder neck and was protruding into the internal urethral meatus (Fig. 3). We discovered that it was attached to the bladder wall. Excision of the bladder mass was performed. During surgery, multiple cystic masses containing dark-brown liquid were excised. The integrity of the urinary bladder wall was restored. The urinary bladder was drained by a size 16 Foley urethral catheter.

After removing the urethral catheter (at 48 h), our patient was free from her symptoms of bedwetting, difficulty in starting the stream of urine, and intermittent interruption of the urinary stream while voiding.

**Fig. 4:**
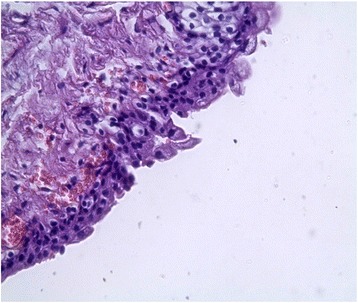
Mucous membrane of the urinary bladder. Cells located parallel to each other, their nuclei light, without mitotic activity. Dysplasia, number of asymmetrically arranged cells seen with enlarged nuclei. Round-cell infiltration is expressed to a greater degree than in typical plasma cells. Polymorphism of the nuclei is weakly expressed. There are some giant forms of nuclei. Within the stroma, thin, full-blooded vessels are seen. Hematoxylin and eosin (× 180)

**Fig. 5:**
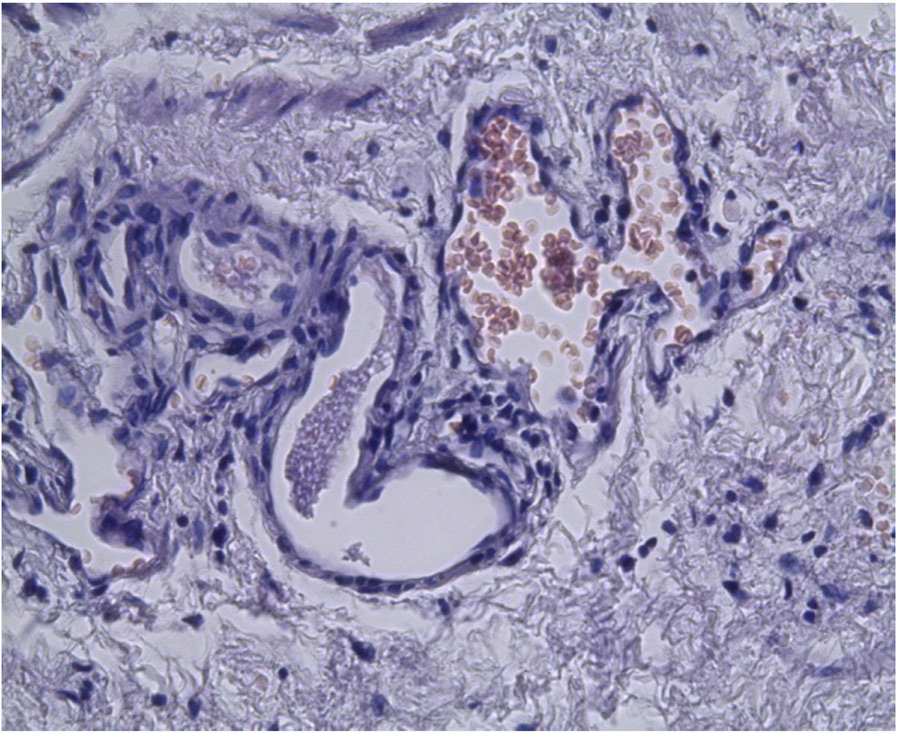
Irregular cavities due to protruding small papillary epithelial outgrowths. Some of the cavities communicate with the mucous membrane through a narrow passage. In some fields of view, a dilated S-shaped bend of the nephron with formation of small cysts under the capsule is seen. Vessels are full-blooded and their lumen dilated. Hematoxylin and eosin (× 180)

**Fig. 6:**
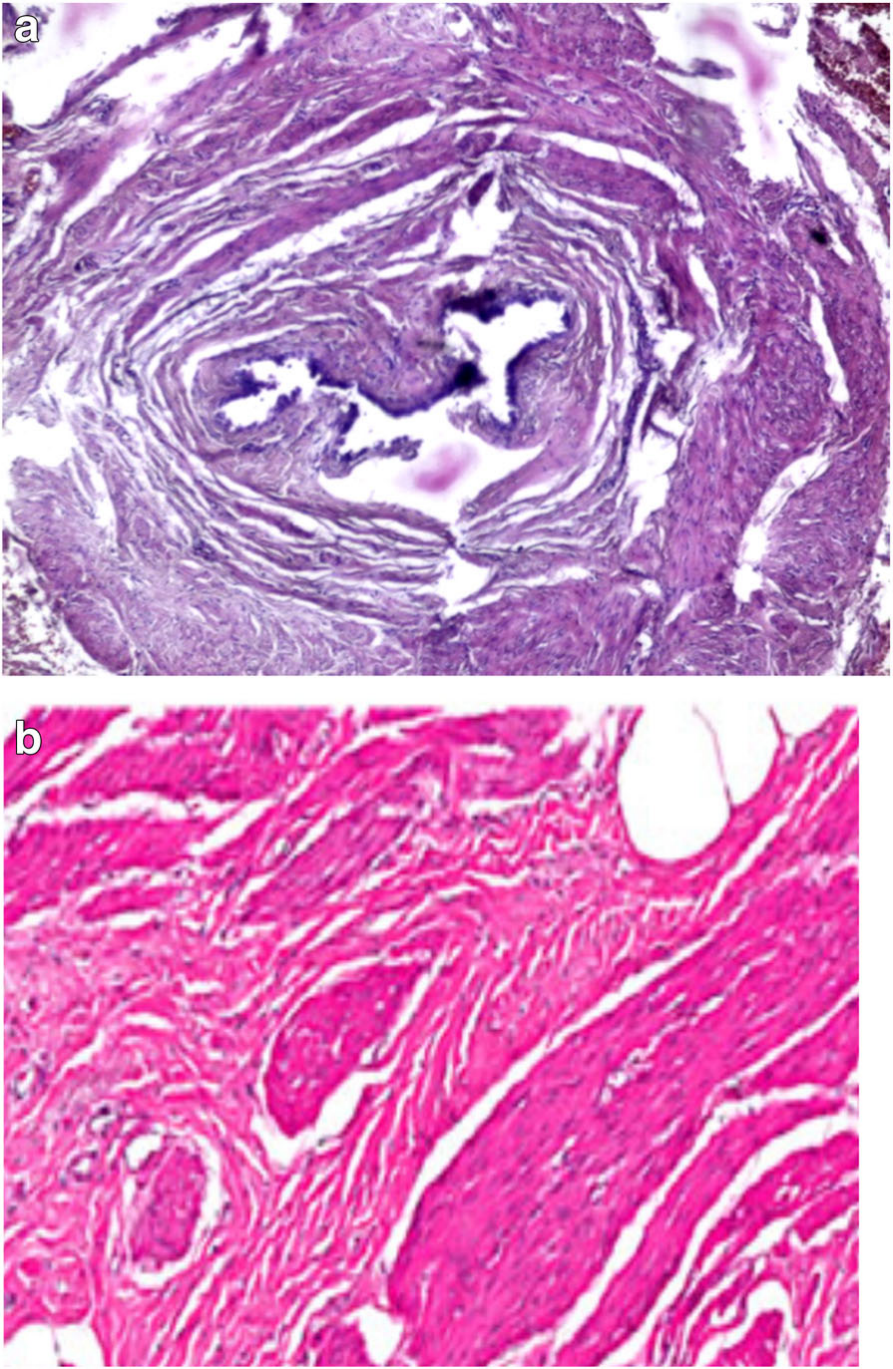
**a** Malformed tubular structure lined with cubic epithelium. **b** Enlarged interstitial tissue. Hematoxylin and eosin (× 180)

**Fig. 7:**
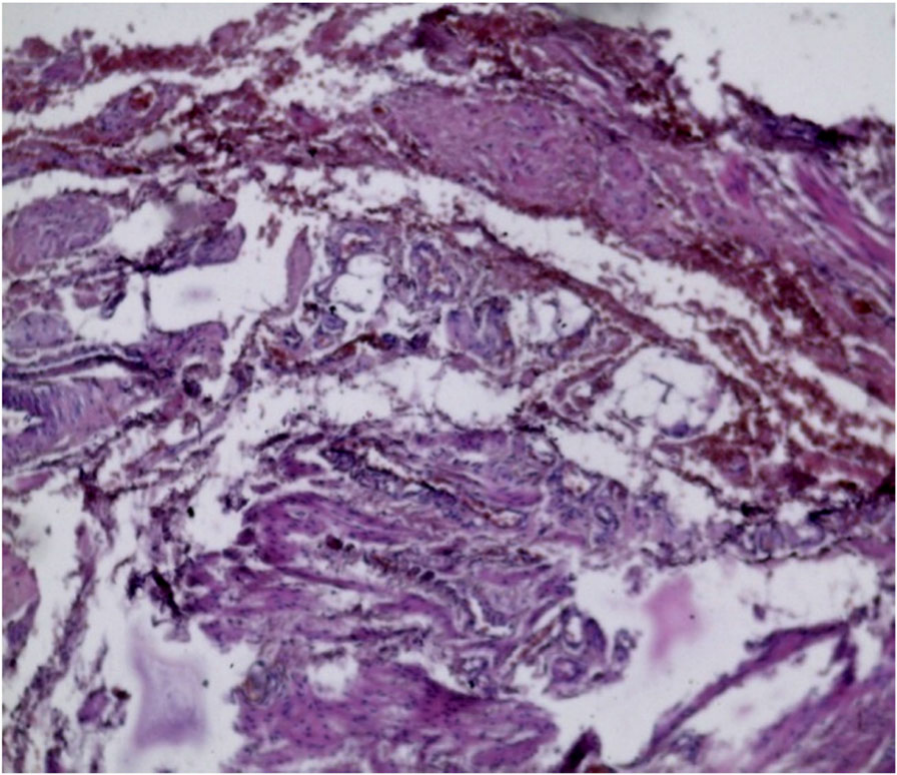
Dilated distal tubules, collecting ducts, and encapsulated cysts. Some of tubules are atrophied. Hematoxylin and eosin (× 180)

**Fig. 8:**
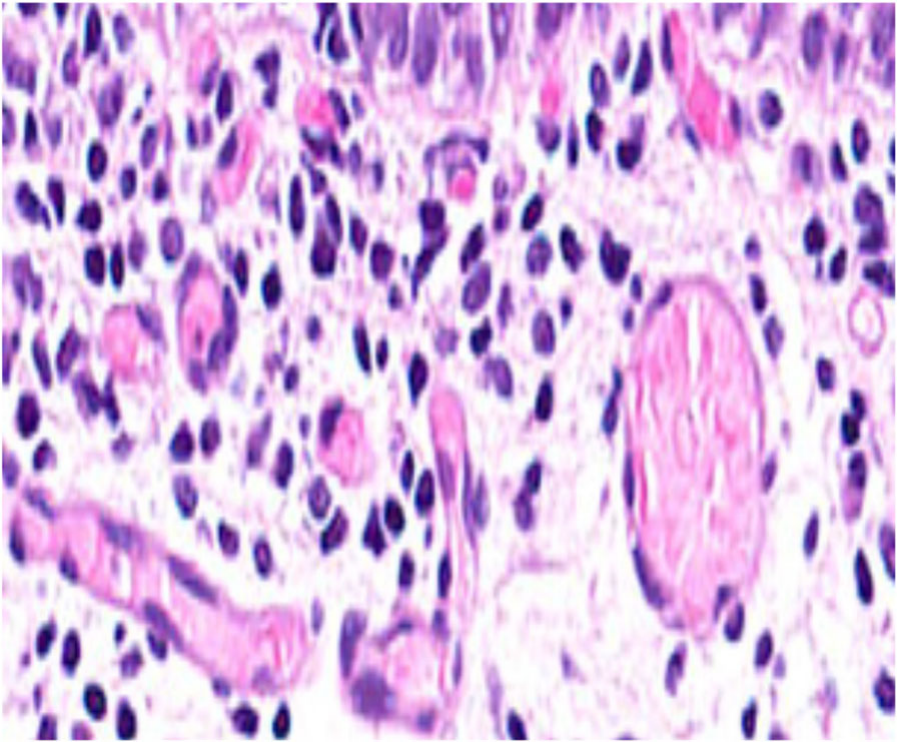
Cystic dilation of tubules and collecting system, some of them curved, also seen are dysplastic cells, enlarged nuclei, and a number of asymmetrically located cells. Round-cell infiltration is expressed to a greater degree than in typical plasma cells. Polymorphism of the nuclei is weakly expressed. There are some giant forms of the nuclei. Hematoxylin and eosin (× 180)

**Fig. 9:**
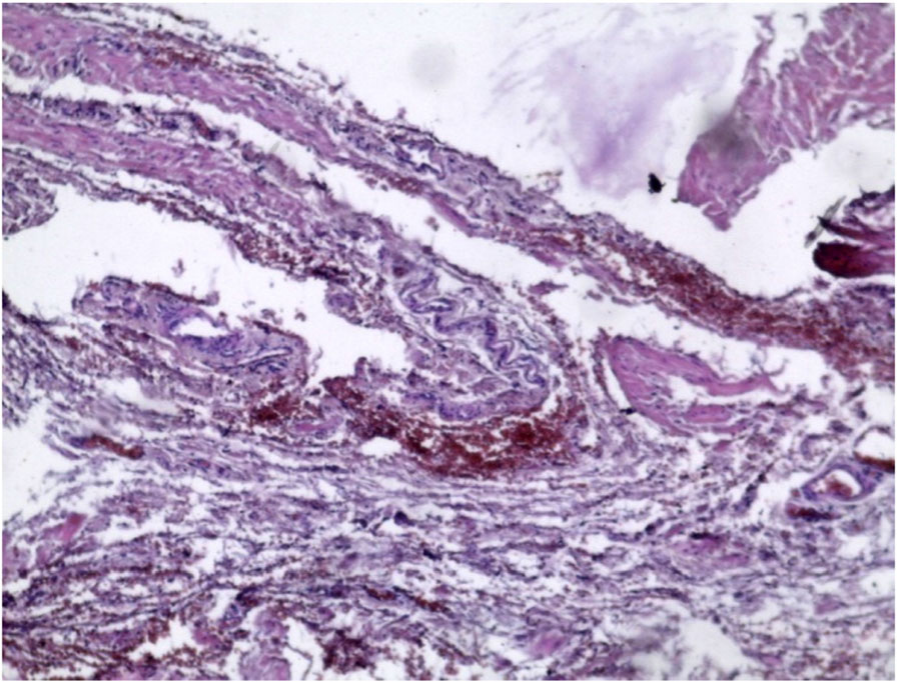
Residual structures of the renal pelvis and calyces; tubular structures in the form of cysts. Hematoxylin and eosin (× 180)

**Fig. 10:**
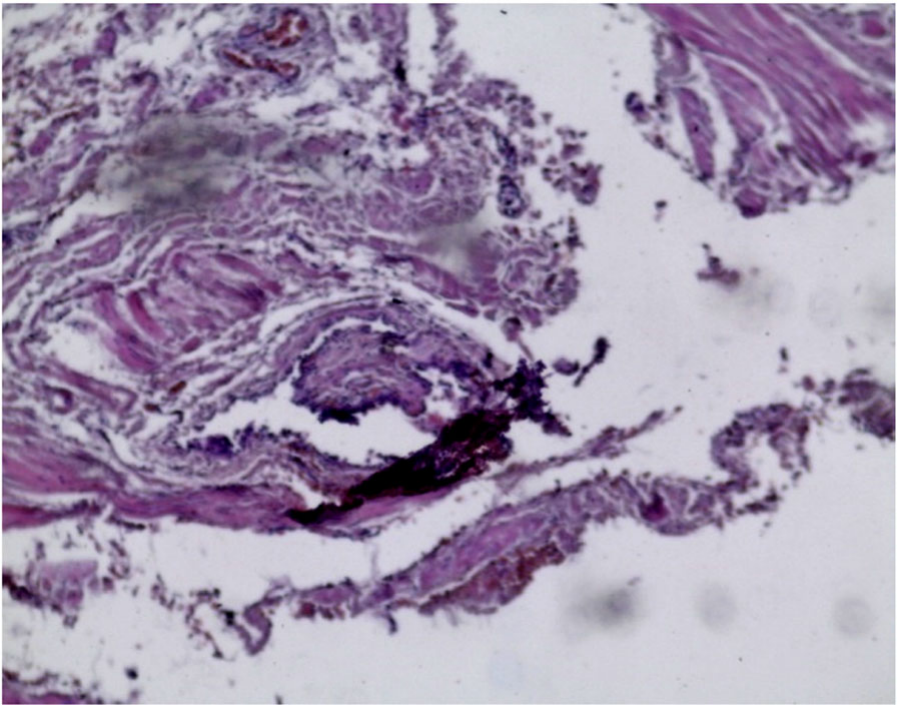
Atrophy of renal tubules. The basement membrane is thickened, resembling a wall of cysts. Hematoxylin and eosin (× 180)

In order to study the tissue with a light microscope, the specimen was fixed in formalin (10%), cut into sections of 5 μm and stained with hematoxylin and eosin. At histology (Figs. 4, 5, 6, 7, 8, 9, and 10), malformation of renal structures typical of rudimentary multicystic dysplastic kidney was observed: a tubular structure lined with cubic epithelium, dilated distal tubules and collecting ducts with their transformation into stretched cysts; disorganized structures of renal calyces and pelvis that transformed into the cysts; atrophy of renal tubules; thickening of the basement membrane, resembling wall of cysts; malformed glomeruli and tubules are lacking proper organization, interstitial and periglomerular fibrosis, and nonspecific cellular infiltration.

**Fig. 11:**
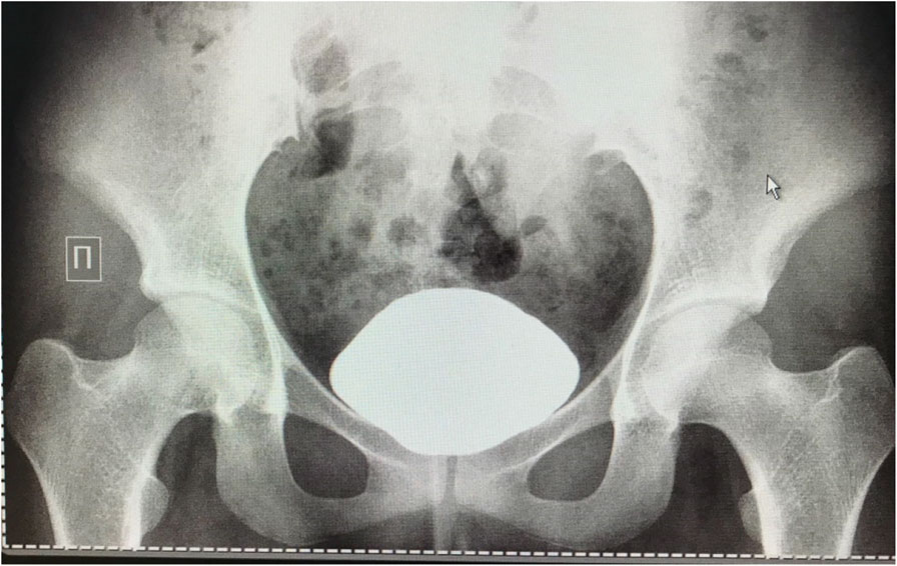
Retrograde cystogram (February 1, 2018). 3 years 8.5 months after surgery (excision of the bladder cystic mass – the cause of the difficulty in starting the stream of urine, intermittent interruption of the urinary stream while voiding, and bedwetting, which turned out to be a pelvic dystopia of right rudimentary multicystic dysplastic kidney). Normal urinary bladder. No cystic mass seen

At 3 year, 8.5 month follow-up of the patient, she had no recurrence of bedwetting, difficulty in starting the stream of urine or intermittent interruption of the urinary stream while voiding. She was followed up with clinical and radiological examinations. Retrograde cystogram (February 1, 2018) showed normal urinary bladder, no cystic mass was seen (Fig. 11).

## Discussion

Usually, most cases of bedwetting are successfully treated by traditional methods of treatment.

We have presented a case of a very rare patient with multiple congenital malformations of the urinary and genital system (Pelvic dystopia of right rudimentary multicystic dysplastic kidney, single pelvic ectopic left kidney, agenesis of the uterus and vagina (Mayer-Rokitansky-Küster-Hauser [MRKH] syndrome), in which pelvic dystopia of right rudimentary multicystic dysplastic kidney caused bedwetting, difficulty in starting the stream of urine, and intermittent interruption of the urinary stream while voiding. We searched Medline, Embase, and the Cochrane Database of Systemic Reviews using the search terms causes of “enuresis” or “bedwetting” as keywords, and found no report about dystopia of rudimentary multicystic dysplastic kidney being the cause of bedwetting.

Usually, most common urological causes of bedwetting are: overactive bladder [3] or dysfunctional voiding cystitis [4], urinary tract infection, urethral obstruction [5], and ectopic ureter [6].

Most studies indicate that children with more severe form of enuresis are more likely to have persistent problems [7] and treatment for these children should not be delayed. Importantly, nocturnal enuresis can be cured and improvement of quality of life has been reported after successful treatment [8]. It is, therefore, important to offer timely treatment and to refer children for qualified specialist care when treatments are not effective.

In 1993, 441 patients with multicystic kidney were registered [9]. Approximately 18% of these kidneys were undetectable by age 1 year, 31% by age 3 years, and 54% by age 5 years. Renal agenesis can occur secondary to a defect of the Wolffian duct, ureteric bud, or metanephric blastema. Unilateral renal agenesis has an incidence of 1 in 450–1000 births; because the Wolffian and Müllerian ducts are contiguous, Müllerian abnormalities in girls are also common; the Mayer-Rokitansky-K**ü**ster-Hauser syndrome (1 in 4000–10,000 female births) is a group of associated findings that may include vaginal aplasia, uterine maldevelopment, and normal ovaries [10, 11]. Unilateral multicystic dysplastic kidney is the most common case of renal cystic disease in children, and malformations of the contralateral urinary tract and kidney (ureteropelvic obstruction, megaureter, reflux, renal dysplasia) have been reported [12]. Macrocysts in multicystic dysplastic kidney appear obvious only in the early third trimester of pregnancy. After reaching a maximum size, the cysts start to involute either in utero or after birth, which may lead to a small noncystic mass, so-called aplastic kidney, or even to complete disappearance of the entire dysplastic kidney. The multicystic dysplastic kidney is a progressive and changing disorder [13]. The metanephric kidney is initially sacral, as the outgrowth lengthens, it becomes positioned more and more cranially [14]. But in our case, dysplastic kidney was not in ascent and remained in its metanephric state on the sacral level, as proven by our imaging studies, surgical procedure, and histologic examination.

However, the literature does not describe cases where the pelvic dystopia of rudimentary multicystic dysplastic kidney was a cause of bedwetting, which makes our case a rare one.

## Conclusions

When conventional methods of treatment of nocturnal enuresis in children are ineffective, other causes which require surgical intervention should be excluded. One of the very rare causes may be dystopic rudimentary multicystic dysplastic kidney, which can be successfully treated by transvesical excision.

In such difficult cases, where radiologic and endoscopic findings (bladder mass) mislead clinicians with ureterocele, papilloma, or mixed tumor of the urinary bladder, collaboration between surgeons and pathologists is very important when accurate diagnostic search work is required.

## References

[1] National Institute for Health and Care Excellence. Bedwetting in under 19s: NICE Clinical Guideline. October 2010. 39808006

[2] Butler RJ, Golding J, Northstone K. Nocturnal enuresis at 7.5 years old: prevalence and analysis of clinical signs. BJU Int. 2005;96(3):404–10. 10.1111/j.1464-410X.2005.05640.x 16042739

[3] Kawauchi A, Tanaka Y, Naito Y, Yamao Y, Ukimara O, Yonada K, *et al*. Bladder capacity at time of enuresis. Urology. 2003;61:1016–8. 10.1016/s0090-4295(02)02515-3 12736027

[4] Schultz-Lampel D, Steuber C, Hoyer PF, Bachmann CJ, Marschall-Kehrel D, Bachmann H. Urinary Incontinence in Children. Dtsch Arztebl Int. 2011;108(37):613–20. 10.3238/arztebl.2011.0613 PMC3187617 21977217

[5] National Institute of Diabetes and Digestive and Kidney Diseases. Definition and facts for bladder control problems and bedwetting in children. Natl Inst Diab Digest Kidn Dis. 2017.

[6] Senel U, Tanriverdi HI, Ozmen Z, Sozubir S. Ectopic ureter accompanied by duplicated ureter: three cases. J Clin Diagn Res. 2015;9(9):PD10–2. 10.7860/JCDR/2015/13933.6446 PMC4606278 26500949

[7] Butler RJ, Heron J. The prevalence of infrequent bedwetting and nocturnal enuresis in childhood: a large British cohort. Scand J Urol Nephrol. 2008;42:257–64. 10.1080/00365590701748054 18432533

[8] Redsell SA, Collier J. Bedwetting behavior and self-esteem: a review of the literature. Child Care Health Dev. 2001;27:149–62. 10.1046/j.1365-2214.2001.00195.x 11251613

[9] Wacksman J, Phipps L. Report of the Multicystic Kidney Registry: preliminary findings. J Urol. 1993;150(6):1870–2. 10.1016/s0022-5347(17)35918-9 8230521

[10] Elder JS. Congenital anomalies and dysgenesis of the kidneys. In: Kliegman R, Stanton B, St. Geme J, Schor NF, Behrman RE, editors. Nelson textbook of pediatrics, Book 2. 20th ed. Philadelphia: Elsevier; 2015. p. 2554.

[11] Fontana L, Gentilin B, Fedele L, Gervasini C, Miozzo M. Genetics of Mayer-Rokitansky-Küster-Hauser (MRKH) syndrome. Clin Genet. 2017;91(2):233–46. 10.1111/cge.12883 27716927

[12] Kuwertz-Broeking E, Brinkmann OA, Von Lengerke HJ, Sciuk J, Fruend S, Bulla M, Harms E, Hertle L. Unilateral multicystic dysplastic kidney: experience in children. BJU Int. 2004;93(3):388–92. 10.1111/j.1464-410x.2003.04623.x 14764144

[13] Avni EF, Thoua Y, Lalmand B, Didier F, Droulle P, Schuman CC. Multicystic dysplastic kidney: natural history from in utero diagnosis and postnatal follow-up. J Urology. 1987;138(6):1420–4. 10.1016/s0022-5347(17)43660-3 3316716

[14] Patten BM. Human embriology. 3rd ed. New York: McGraw-Hill; 1968. p. 449–99.

